# Two Cases of Orbital Myositis as a Rare Feature of Lyme Borreliosis

**DOI:** 10.1155/2011/372470

**Published:** 2011-07-28

**Authors:** Arnaud Sauer, Claude Speeg-Schatz, Yves Hansmann

**Affiliations:** ^1^Service d'Ophtalmologie, Hôpitaux Universitaires de Strasbourg, Nouvel Hôpital Civil, BP 426, 67091 Strasbourg Cedex, France; ^2^Service de Maladies Infectieuses et Tropicales, Hôpitaux Universitaires de Strasbourg, Nouvel Hôpital Civil, BP 426, 67091 Strasbourg Cedex, France

## Abstract

Myositis has been reported as a rare manifestation of Lyme disease, and the Lyme disease spirochetes can be an important consideration in the differential diagnosis of unusual cases of myositis, especially in patients who live in or travel to endemic areas. We report the case of two patients who presented with focal orbital myositis which are rare localization for Lyme disease. Myositis were confirmed by magnetic resonance imaging. Diagnosis criteria for *Borrelia burgdorferi (B. burgdorferi)* infection was supported by (i) medical history (tick bite in an endemic area), (ii) systemic clinical findings (*Erythema migrans*, neurological manifestation or arthritis), (iii) positive Lyme serology and/or the detection of *B. burgdorferi* DNA by polymerase chain reaction, as well as (iv) exclusion of other infectious and inflammatory causes. The current cases are reviewed in the context of findings from previous myositis descriptions.

## 1. Introduction

Myositis has been reported as a rare manifestation of Lyme disease, and the Lyme disease spirochetes can be an important consideration in the differential diagnosis of unusual cases of myositis, especially in patients who live in or travel to endemic areas. Lyme borreliosis can also cause a variety of ocular manifestations (papillary edemas or abducens palsy are among the most frequent), and the frequency of these manifestations among cases of Lyme disease involving systemic manifestations is about 1%. We report the case of two patients who presented with acute diplopia and orbital swelling secondary to focal orbital myositis which are very rare localization for Lyme disease. Myositis was confirmed by magnetic resonance imaging. Diagnosis criteria for* Borrelia burgdorferi* (*B. burgdorferi)* infection were supported by the association of five criterions, (i) medical history (tick bite in an endemic area), (ii) systemic clinical findings (*Erythema migrans*, arthritis), (iii) lab confirmation by positive Lyme serology, (iv) exclusion of other infectious and inflammatory causes, and (v) efficacy of specific antibiotherapy. The current cases are reviewed in the context of findings from previous myositis descriptions.

## 2. Cases Report

We report on the clinical findings in two patients (2 females: 13 and 68 years old) with isolated orbital myositis. The two patients had taken a medical advice for acute unilateral orbital pain and diplopia.

**Figure 1:**
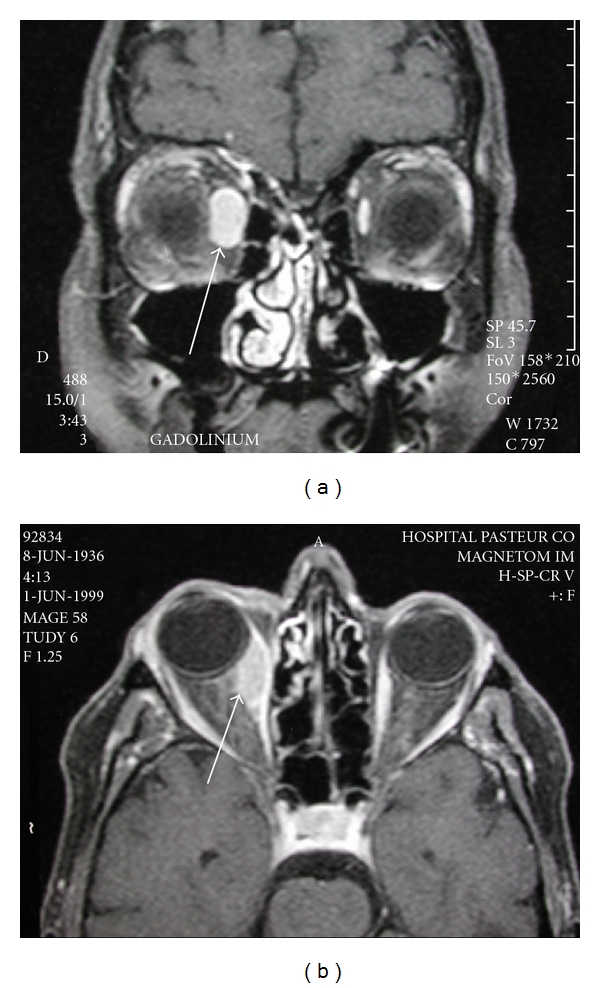
High signal intensity on MRI images in an orbital myositis related to Lyme disease.

The 68-year-old patient had acute and recurrent episode (lasting from 2 to 4 weeks, 2 to 4 times each year) of right orbital swelling and pain since three years. Each episode resolved spontaneously or with steroids and nonsteroidal anti-inflammatory drugs. As she complained about a new episode of orbital swelling complicated with horizontal diplopia, a complete workup was done. A high intensity signal on MRI was observed on the right medial rectus muscle, confirming diagnosis of orbital myositis (Figure 1). No systemic disease (such as Grave's disease or sarcoïdosis) or haematological disease or any other infectious disease was found. As she was living in a rural area of Eastern France, a highly endemic country for Lyme disease, and was remembering numerous tick bites and had a history of *erythema migrans* and arthralgia, Lyme disease was suspected. Determination of antibodies related to *Borrelia* was positive. It was decided to begin an antibiotic therapy with doxycyclin (200 mg/day), which allowed resolution of ocular and imaging symptoms within 3 weeks. After a followup of 15 years, no relapse of orbital swelling was noted for her. 

The 13-year-old woman complained about unilateral orbital swelling complicated with exophtalmia and horizontal diplopia. MRI showed a hyperintense signal of right inferior and medial rectus muscles. Because she was living in the same highly endemic area for Lyme disease and had a recent medical history of tick bite followed by *Erythema migrans*, serologic test for testing the presence of *Borrelia *antibody was rapidly done and was positive. Systemic disease (such as Grave's disease, dysthyroid, or sarcoïdosis) or haematological disease or any other infectious disease was excluded. A 4-week treatment with doxycyclin (200 mg/day) was successful: resolution of ocular symptoms and a decrease of the MRI signal intensity were observed within 1 month. After a 14-month followup, there was no relapse of the disease.

## 3. Discussion

We reported the cases of two patients who presented with orbital myositis caused by *Borrelia *infection at (XXX), which is situated in a highly endemic area for Lyme borreliosis. Diagnosis was done on medical history (tick bite that occurred in an area of endemicity), ocular findings, and systemic clinical findings (*erythema migrans* or arthralgia), as previously published [1, 2]. 

Systemic disease, such as Grave's disease, or haematological disorders like lymphomas are the principle cause for orbital myositis. In these aetiology, myositis is often bilateral. Infectious diseases, such as staphylococcal septicaemia, may be another cause for unilateral myositis. The aetiologic research for our two patients did not conduct to any of these causes. Only borrelia serology was positive, suggesting the causality of Lyme borreliosis. So, our patients were treated with antibiotics efficient against *Borrelia*. Then, the evolution was absolutely significative, with regression of clinical and radiological signs of myositis in some weeks, without any anti-inflammatory treatment. Furthermore, for one of the patients, this good evolution under antibiotics treatment occurred even after a long period, whereas corticosteroid therapy did not show any efficacy, arguing for the borrelial origin.

While myalgias are frequently associated with Lyme disease, myositis is a rare manifestation of borrelial infection. Indeed, localized involvement, as in the present cases, is considered to be characteristic of Lyme disease [3]. Borrelial myositis is generally localized to the vicinity of cutaneous lesions [4]. Symptoms and manifestations are protean and include pain, tenderness, swelling, and weakness, as in the present cases. Biological markers such as creatine kinase levels have most often been reported to be normal [3–5]. We have not performed oculomotor muscle biopsy because of the high rate of potential complications and because the aetiological diagnosis was considered as very probable. Moreover, in previous reports, the histopathologic findings were not specific and consisted chiefly of a monocellular, lymphoplasmacytic, and histiocytic infiltrate composed essentially of macrophages and T helper/inducer (CD4+) cells [6]. 

At present, Lyme borreliosis is diagnosed mainly on the basis of clinical symptoms and serological tests. These tests are based on demonstration in human serum of anti-*B. burgdorferi* s.l. IgG and IgM antibodies and are usually carried out by enzyme-linked immunosorbent assay (ELISA) and Western blot confirmation. Unfortunately, serological tests have a weak sensitivity in presence of *Erythema migrans* (20 to 50%), and, conversely, sensitivity is rising during secondary or tertiary phase between 70 and 90%. However, specificity of a positive serology remains weak because of numerous false positive and crossed reactions with other bacteria [7]. Regarding the multiplicity of ocular manifestations of Lyme disease, the rigorous application of diagnosis criterion is imperious to avoid “excess-diagnosis”, as used in former studies [1, 2]: (i) a history of tick bite, (ii) the presence of systemic findings (*erythema migrans*, neurological manifestations, arthritis), (iii) the presence of antibodies related to *Borrelia *species and/or detection of bacterial DNA by polymerase chain reaction, (iv) the exclusion of other infectious and inflammatory condition, and (v) the efficacy of antibiotics therapy. 

Literature features have shown that therapeutic antibiotic regimens were highly varied but usually successful, as in the present cases. These regimens have included treatment with penicillins, cephalosporins, and tetracyclines for durations varying from 10 days to 2 months. Corticosteroids have not infrequently been used as part of the treatment strategy [1, 2, 8, 9].

Orbital myositis is an unusual manifestation of Lyme disease, although it is likely that the condition is underdiagnosed. Unexplained muscle swelling occurring in a patient who has had a rash or a recent history of a tick bite in an endemic area for Lyme disease should prompt consideration of this diagnosis. The diagnosis can usually be made on the basis of clinical features and serologic studies. MRI may be a useful adjunct for diagnosis and followup.
